# T-regulatory cell modulation: the future of cancer immunotherapy?

**DOI:** 10.1038/sj.bjc.6605040

**Published:** 2009-04-21

**Authors:** S Nizar, J Copier, B Meyer, M Bodman-Smith, C Galustian, D Kumar, A Dalgleish

**Affiliations:** 1 Department of Cellular and Molecular Medicine, St George's University of London, London, UK; 2 Department of Colorectal Surgery, St Georges Hospital NHS Trust, UK

**Keywords:** T-regulatory, tregs, immunotherapy

## Abstract

T-regulatory cells suppress anti-tumour immunity in cancer patients and in murine tumour models. Furthermore, their activity is likely to have an effect on the effectiveness of immunotherapeutic treatments for cancer. Here we describe the current status of developing clinical strategies for modulating Treg activity in cancer patients.

A strong relationship exists between T-regulatory cells (Tregs) and the development and progression of cancer. Tregs within the tumour, ascites and peripheral blood of patients with cancer are associated with poor prognosis. Increasingly, evidence suggests that Tregs protect tumours from the potentially effective immune responses. Thus, new anti-cancer strategies involving interference in Treg biology or depletion of Tregs are of critical importance. Among the plethora of agents known to affect Tregs, there are some conventional therapeutic agents. Here we describe the current status of clinical modalities, both specifically designed to target Tregs and those drugs for which their immunomodulatory function may constitute a hitherto undefined mode of action.

## A brief history of tregs

Suppressor cells, first observed in the 1970s, were thought to be antigen-specific and, once activated, targeted CD4+ T-helper cells, blocking activation and progression of both humoral and cell-mediated immunity. Subsequently, the mechanism of suppression was found to be more complicated and, because of the lack of any phenotypic marker, the term T-‘suppressor’ cells drifted out of use. More than 20 years later, CD4+ T cells expressing interleukin (IL)-2 receptor *α*-chain (CD25) that maintained tolerance against auto-immune diseases in mouse models were identified (Sakaguchi *et al*, 1995). These ‘Tregs’ make up 5–10% of naïve CD4+ T cells in the periphery, and seem to be a heterogeneous group of CD4+ cells variable in their mechanism of action.

The Treg subtypes that are affected by the treatments discussed in this review are unclear in many cases. In the following section a brief overview of Tregs is intended to introduce key cell types that may play a role in tumour biology. A complete review of Treg biology is beyond the scope of this paper, and has been extensively reviewed elsewhere.

The thymus-derived Tregs or natural Tregs (nTregs) are easily identified by CD4 and high CD25 expression. However, differentiating between CD4+ T cells and human Tregs cells is difficult, as over a quarter of human T cells express CD25 to some degree. Recently, FOXP3 (Forkhead box P3 transcription factor) has helped to differentiate the suppressor population of CD4+ CD25+ cells. Initially identified as a mutated gene in the *scurfy* mouse strain, which developed autoimmunity because of CD4+ T-cell hyperactivity and increased production of pro-inflammatory cytokines, FOXP3 was found to be a key regulatory gene for Tregs. The exact mechanism of nTreg suppression is not clearly defined, but is thought to be involved in cell-to-cell contact through membrane-bound TGF-*β* (seen in certain *in vitro* studies), cytokine release (IL-10, TGF-*β*, IL-35), signalling through cAMP and possibly extracellular adenosine. It has been difficult to identify an all-encompassing suppressive pathway as yet in *in vivo* studies and in human studies in particular.

Inducible Tregs are very similar in function to nTregs but derive from FOXP3 negative naïve T cells in the periphery under specific stimulation. Once induced these cell begin to express FOXP3, cytotoxic T-lymphocyte antigen-4 (CTLA-4), and secrete IL-10 and TGF-*β*.

Tr1 cells are adaptive Tregs that differ from nTregs in their ability to produce the IL-10 and TGF-*β* in large amounts, which both suppress naïve and memory CD4+ T-cell function in murine *in vitro* studies (Groux *et al*, 1997). Tr1 cells are also induced by antigen-mediated TCR activation in the presence of IL-10 (Roncarolo *et al*, 2006).

Th3 cells (another adaptive Treg subset vital for the maintenance of oral tolerance) achieve their suppressive effects through the secretion of IL-10, IL-4 and large amounts of TGF-*β*, and indirectly by promoting the differentiation of antigen-specific FOXP3 Tregs peripherally. Th3 cells differ from Tr1 in their dependence on TGF-*β* for differentiation from CD4+CD25–T cells (Carrier *et al*, 2007).

A diverse group of T cells with regulatory properties exists. As yet, not all have been identified and some may well be the same cell at different stages of activation. In fact, when treating patients it is difficult to discern the type of Treg that is being targeted, as the main mode of Treg evaluation is the peripheral blood sampling (in which FOXP3 seems to be the main identifier) and basic functional analysis. Inducible Tregs and natural Tregs seem to be the important cell types in immune regulation in cancer patients. One particular murine *in vivo* study suggests that conversion of naïve CD4+ T cells into antigen-specific iTregs takes place within the tumour microenvironment, and is driven by the tumour itself (Zhou and Levitsky, 2007). At present the specific contributions of the different subtypes within the tumour milieu is not certain.

## Tregs and malignancy

T-regulatory cells are implicated in the development of autoimmunity, allergy and rejection of organ transplants, as well as the suppression of immune responses to cancer. There is an increased presence of CD4+CD25+ T cells in a wide spectrum of human malignancies, such as lung, head and neck, ovarian, gastrointestinal and skin. These cells are found in relatively high concentrations in blood, ascites, tumour draining lymph nodes and within the tumour milieu of cancer patients. Increased accumulation of Tregs conferred a poor prognosis for patients with ovarian cancer (Curiel *et al*, 2004). However, several recent studies showed conflicting prognostic data for some haematological, specifically B-cell lymphoma, malignancies in which higher numbers of FOXP3+ cells (taken to be Tregs) were shown to correlate with improved survival. Curiously, in B-CLL it has been shown that high Treg numbers also correlate with advanced disease stage. The differences in prognostic benefit of Tregs between haematological malignancy and carcinoma remain to be clarified. However, it has been suggested that the earlier shown suppressive effects of Tregs on B-cell function may explain the benefit of high Treg numbers in B-cell lymphoma patients (Tzankov *et al*, 2008). It is worth adding that many of these studies concentrate only on the numbers or percentages of FOXP3+ cells and, although this clearly identifies Tregs, it is our experience that many tumour cells also express FOXP3, lending the possibility that not all cells identified as FOXP3 positive are, in fact, Tregs.

Much research has been undertaken to determine how Tregs help tumours escape detection and elimination. Tregs affect many of the immune cells that reside in the tumour milieu and draining lymph nodes, including the effector T cells, natural killer cells and dendritic cells. These cells are paramount for effective cancer immunity and thus would explain the role of Tregs, which accumulate in large numbers.

## Impact of chemotherapy

Modulating the action of Tregs represents one aspect in the prevention of tumour ‘immune’ escape, which potentially enhances immunosurveillance and the effects of other immunotherapeutic modalities. A number of agents affect Tregs in a clinical context, some of which are designed specifically to target known receptors on Tregs, whereas others, including conventional chemotherapeutic drugs, have modulatory effects on Tregs although their exact mode of action is often unknown.

Some commonly used chemotherapeutic agents, such as methotrexate and cyclophosphamide, can have immunostimulatory and anti-angiogenic effects at lower doses without the toxic effects associated with higher doses. This new concept of treatment is on the basis of the more frequent or ‘metronomic’ administration of a dose substantially lower than the maximum tolerated dose. Cyclophosphamide depletes CD4+CD25+ Tregs in mice injected with tolerogenic syngeneic tumour cells (Ghiringhelli *et al*, 2004). Depletion leads to a decrease in tumour cell growth and eventual rejection of established tumours when used in combination with an immunotherapeutic agent (PROb tumour cells mixed with BCG). Cyclophosphamide not only depletes Treg numbers by increasing their susceptibility to apoptosis, but also has a deleterious effect on their function in a murine *in vitro* study. There is some evidence that the negative effect of low-dose cyclophosphamide is attributable to a decreased expression of glucocorticoid-induced tumour necrosis factor receptor (GITR) and FOXP3. The restoration of Treg numbers and the function 10 days after cyclophosphamide administration indicates the reversibility of its effects (Lutsiak *et al*, 2005).

Low-dose cyclophosphamide has been used in a metronomic regimen in patients with end-stage cancer (Ghiringhelli *et al*, 2007) and conventional hormone-resistant prostate cancer (Lord *et al*, 2007), potentiating its anti-angiogenic and immunostimulatory properties and avoiding its high-dose toxic effects. Though the phase II prostate cancer study showed no relationship between clinical response and circulating Treg numbers, there was a reduction in TGF-*β* expression, which is crucial for nTreg-mediated suppression. The study of nine patients with metastatic end-stage cancer treated with metronomic low-dose cyclophosphamide (50 mg orally, once daily for a week with the ensuing week off) did, however, show a selective reduction in T-regulatory cell numbers with a preservation of total number of lymphocytes and natural killer cells. Contrary to the evidence provided thus far, one recent French study evaluating varying doses of cyclophosphamide showed neither improved clinical benefit, nor concomitant decrease in peripheral Treg numbers, nor any decline in FOXP3 expression (Audia *et al*, 2007). However, in contrast to the continuous low-dose regimen, they administered a single bolus dose. Moreover, histological analysis of tumours from five patients suggested a reduction in CD25+ T-cell aggregation in the tumour vicinity and increased infiltration of CD8+ T cells, which hints at an immunostimulatory benefit.

A number of other chemotherapeutics used in standard practice modulating Tregs, include gemcitabine, mitoxanthrone, fludarabine and COX-2 inhibitors. Although Treg modulation is by no means the primary mode of action for these drugs, it is interesting to speculate that these, and potentially other standard chemotherapeutics, have secondary functions mediated through their effects on Tregs.

Gemcitabine, a nucleoside analogue, used in pancreatic cancer inhibits DNA synthesis in the S-phase of the cell cycle. Gemcitabine has a mixed effect on the peripheral blood lymphocytes. When administered to patients with non-small cell lung cancer in a phase I study, lymphopenia with particular decrease in effector T-cell populations was observed (Levitt *et al*, 2004). The pancreatic cancer patients receiving gemcitabine had an initial reduction in absolute lymphocyte number, which eventually stabilise (Plate *et al*, 2005). There was no observable reduction in CD25+ T-cell number, but an increase in naïve T-cell activation was observed. In a phase I study of colon cancer patients, there was a rise in CTLs with a concomitant decrease in CD25+CD4+T cells in clinical responders (Correale *et al*, 2005). Despite the favourable objective clinical response across the three studies, the effect of gemcitabine on lymphocyte populations is mixed. In a phase I study in lung cancer, there was a profound effect on the rapidly dividing cells including lymphocytes. It is interesting that Gemcitabine was able to eliminate myeloid-suppressor cells (MSCs), which also have immunosuppressive properties, in a murine study leading to increased activation of CD8+ T cells (Suzuki *et al*, 2005). Gemcitabine has broader effects on a variety of suppressive cells, and the clinical effect seen in some studies evaluating Tregs may be partially related to the elimination of MSCs. Further work is needed to define its mechanism of action.

Mitoxantrone is an anthracenedione that binds to deoxyribose on DNA causing strand break up and unravelling. When administered to patients with breast cancer, an objective tumour response is seen in approximately one-third of the participants. Though there was no change in the CD4/CD8 ratio, there was a significant drop in B lymphocytes, T-‘suppressor’ cells and CD25+ cells (Barni *et al*, 1991). Despite this depletion there was no observable relationship with the tumour response. Thus mitoxantrone, although ineffective in breast cancer as a single modality, may be a suitable agent for combined use with immunotherapy, which may have clinical benefit in breast cancer.

Fludarabine, which has good efficacy in the treatment of chronic lymphocytic leukaemia, has the capability to inhibit Treg expansion and maintains CTL function in co-culture studies (Hegde *et al*, 2008). Though patients treated with fludarabine have a general reduction in CD4+ T cells, in a study where patients with CLL were treated with fludarabine a preferential decline in CD4+CD25+ Tregs was noted due to apoptosis (Beyer *et al*, 2005). An optimum dose must be determined that will safeguard effector T-cell function if fludarabine is to be used in an immunomodulatory role.

COX-2 expression is closely allied to many aspects of tumour progression. Patients taking aspirin and other nonsteroidal anti-inflammatory drugs have significant protection from developing colorectal cancer. *In vitro* tumour-derived prostaglandin E_2_ (PGE_2_) increases FOXP3 expression and Treg inhibitory activity. COX-2 inhibition significantly reduces tumour Treg infiltration in murine studies. There are increased concentrations of PGE_2_ in the peripheral blood of patients with colorectal cancer, and *in vitro* analysis showed that Indomethacin (a COX-2 inhibitor) reverses Treg-mediated anti-tumour suppression (Yaqub *et al*, 2007). In a recent clinical study, patients with colon cancer were randomised to either an oral NSAID (indomethacin or celebrex) or a control drug (esomeprazole) for 3 days preoperatively. Histological analysis of resected specimens showed a significant increase in CD8+ tumour-infiltrating T-cells and decreased expression of FOXP3 and IL-10 in patients who took indomethacin (Lonnroth *et al*, 2008). In preclinical models COX-2 inhibitors, for example celecoxib, enhance the effects of dendritic cell vaccines. Although celecoxib clearly has effects when delivered as a single modality, it is interesting to speculate that some of the effects of COX-2 inhibition may be because of the suppressive effects on Tregs.

## Direct targeting of tregs

A number of recently developed reagents target Tregs directly, either through recognition of CD25 or through CTLA-4 blockade. Both approaches lack specificity, as they target essential immunoregulatory molecules on T-effector cells. However, this reflects the difficulty of differentiating between Tregs and other effector cells.

CD4+CD25+ Treg depletion by administration of anti-CD25 antibodies in mice produced a significant increase in anti-tumour activity, along with an increased incidence of autoimmune diseases. Multimodal immunotherapy using monoclonal antibodies and vaccines has also been studied in murine models, in which the Treg inhibition leads to an improved effector cell response to the vaccine. More recently, there has been an increased use of recombinant toxic proteins designed to target T cells with high CD25 expression (but spares T lymphocytes not involved in immune suppression).

LMB-2 is a fusion protein consisting of a single-chain Fv fragment of a CD25-specific monoclonal antibody attached to a 38 kDa fragment of *Pseudomonas* exotoxin A. In a preclinical *in vitro* study in which human PBMCs were incubated with LMB-2, CD4+CD25+Tregs were selectively depleted (Attia *et al*, 2006). A phase I study LMB-2 in CD25+ T-cell malignancies showed one complete and seven partial responses among a group of 20 patients receiving more than 60 *μ*g kg^–1^ per cycle with acceptable minor toxic effects (Kreitman *et al*, 2000). Administration of LMB-2 followed by peptide vaccination in patients with melanoma showed a steep drop in FOXP3+CD4+CD25+ Tregs. Despite a lack of objective clinical response, these preliminary data are encouraging and provides a method for Treg elimination (Powell Jr *et al*, 2007b).

Denileukin diftitox (DAB_389_IL-2, Ontak, Seragen Incorporated, Hopkinton, MA, USA) is a fusion protein combining human IL-2 and an enzymatically active domain of diphtheria toxin. It binds preferentially to cells expressing the high-affinity IL-2 receptor (CD25, CD122 and CD132) and is internalised ultimately leading to cell death. Diftitox is efficacious in certain types of T-cell lymphoma with high CD25 expression (Talpur *et al*, 2006) (see Table 1). Diftitox administration in advanced renal cell carcinoma (RCC) patients before dendritic cell vaccination resulted in a reduction in the peripheral Tregs and Treg-mediated immunosuppression. This effect on solid tumours was further corroborated in melanoma patients for whom enhanced antigen-specific CD8 T-cell activity was seen (Mahnke *et al*, 2007). However, a study evaluating the treatment of melanoma patients with diftitox not only failed to show any favourable clinical improvement, but also showed no change in number or suppressive properties of peripheral Tregs (Attia *et al*, 2005a). This remains a controversial area and awaits further data to resolve the discrepancy.

IL-2 plays an important role in T-cell biology and is effective in the treatment of certain cancers, such as RCC and melanoma, where it is associated with increased effector T-cell responses (Rosenberg *et al*, 1994). Tregs are induced by the administration of therapeutic amounts of IL-2. Thus administration of IL-2 to ovarian cancer patients resulted in an increased FOXP3+ Treg proliferation, which was inversely proportional to the number of Tregs present before treatment. However, there was a drop in Tregs in responders to IL-2 once treatment was discontinued (Wei *et al*, 2007). A similar increase in Treg number and function occurred when patients with RCC and melanoma were treated with IL-2 (Ahmadzadeh and Rosenberg, 2006). The tumour regression seen on administration of IL-2 may be associated with concomitant activation of tumour-specific T cells. Clearly the relationship between effector function and Treg numbers requires greater understanding.

Depletion of Tregs may provide a beneficial state in effective immunotherapy. However, depletion of CD25+ Tregs in metastatic melanoma (MM) patients treated with IL-2 resulted in the rapid reaccumulation of suppressive CD25+FOXP3+ Tregs in the periphery (Powell Jr *et al*, 2007a). No objective tumour regression was seen in these patients. Depletion of Tregs by diftitox in RCC patients resulted in a refractory repopulation of 75% of the original number within 2 months in another study (Dannull *et al*, 2005). This rapid reversal indicates the need for serial treatment doses with its ensuing side effects to maintain low Treg numbers. Future strategies should consider this possible refractory accumulation of Tregs, which could attenuate clinical responses.

Cytotoxic T-lymphocyte antigen-4 is found in high levels on CD4+CD25+ Tregs where it suppresses activation and proliferation by binding to CD80/CD86 receptors on effector T cells. The importance of CTLA-4 activation in T-cell homoeostasis was shown in a murine study in which CTLA-4-deficient mice died after massive lymphoproliferation leading to multi-organ failure. Thus, CTLA-4 blockade provides a mode of Treg modulation. The effect of anti-CTLA-4 antibodies on solid tumours was initially recognised in mice in the late 1990s. Other animal studies showed the potency of anti-CTLA-4 antibodies against poorly immunogenic tumours when used with other modes of treatment. Anti-CTLA-4 administered alone can be effective against transplanted and spontaneous tumour models in mice. Antibody therapy has reached early human clinical trials. At present, two humanised anti-CTLA-4-blocking antibodies, MDX-010 (Ipilimumab) and CP-675 206 (Tremelimumab**),** have been used in phase I and II trials.

In a phase I trial treating MM, 36% of patients showing grade III/IV autoimmune toxicity had tumour regression compared with 5% in patients without autoimmune symptoms (Attia *et al*, 2005b). A further trial combining varied doses of MDX-010 with high-dose IL-2 showed synergy compared with earlier studies evaluating IL-2 alone in MM (Maker *et al*, 2005b). Further analysis of the peripheral blood mononuclear cells in patients undergoing anti-CTLA-4 treatment for stage IV MM and RCC has highlighted (by means of *in vitro* co-culture proliferation assay and FOXP3 expression) that there is no inhibition of the suppressive activity of CD4+CD25+ T cells, but a probable enhancement of effector cell function (Maker *et al*, 2005a) (see Table 2).

Tremelimumab is less extensively studied. *In vitro* studies show enhanced T-cell activation. A phase I trial undertaken to establish dosing for phase II concluded that a single dose up to 15 mg kg^–1^ is sufficient to break peripheral tolerance. Out of 39 patients with advanced malignancy two patients had a complete response and two had a partial response. Tremelimumab was shown not only able to suppress Treg activity, but also to replenish the effector and memory CD4+ and CD8+ T-cell numbers, contributing to its anti-tumour effect (Menard *et al*, 2008). Thus, it has been argued that depletion of Tregs may be secondary in importance to modulating the ratio of CD8^+^ effector cells to Tregs, which may be effected through the influence of anti-CTLA-4 antibodies on effector cell numbers. Further phase III studies to evaluate the response to Tremelimumab on patients with MM are planned, and studies on colorectal and lung cancer patients are currently being completed (Cranmer and Hersh, 2007). One phase III randomised study comparing tremelimumab as a first-line treatment with chemotherapy in patients with MM failed to show any improvement in survival and was abandoned after three treatment-related deaths.

These antibodies may well enhance the effectiveness of cancer vaccines when used in combination with other modalities. Sadly, studies to determine the effect of combining GVAX whole-cell vaccine technology for prostate cancer with tremelimumab are likely to be shelved after the failure of phase III studies of GVAX *vs* docetaxel. Further clinical studies will be required in the future if we are to substantiate the use of anti-CTLA-4 therapy in combination with vaccines.

Despite these setbacks there is still much excitement in the recent literature regarding the potential of CTLA-4-blocking antibodies. However, it is important to bear in mind that there is ‘heterogeneity’ in the kinetics of clinical response, with beneficial responses taking place a long time after the administration and unexplained patterns of disease progression (Saenger and Wolchok, 2008). Blockage of CTLA-4 is not complication free with the development of a spectrum of immune-related adverse effects. These side effects will be more frequent with increasing administration of anti-CTLA-4 treatment.

## Future strategies for treg modulation

Targeting glucocorticoid-induced tumour-necrosis factor (TNF)-related peptide receptor is another avenue for modulation of Tregs. GITR is constitutively expressed by Tregs, and when stimulated by either agonist antibodies (DTA-1) to GITR or GITR ligand (Kim *et al*, 2003) leads to suppression of Treg cell activity in murine studies. It is also expressed, albeit at lower levels, on CD4+ and CD8+ T-effector cells and when stimulated results in enhanced proliferation and possible resistance to Treg-mediated suppression. DTA-1 when injected into mice bearing B16 melanoma (a weakly immunogenic tumour) resulted in concomitant immunity and rejection of secondary tumour implantation along with protection against the primary tumour (Turk *et al*, 2004). This shows the importance of Treg attenuation in the presence of a weakly immunogenic tumour and the development of concomitant immunity against melanoma implantation.

In a different model, injection of DTA-1 8 days after the inoculation of BALB/c mice with a methylcholanthrene-induced fribrosarcoma produced tumour-specific immunity (Ko *et al*, 2005). The tumours were infiltrated by large numbers of CD4+ and CD8+ effector cells, and an increase in INF-*γ* was noted. DTA-1 also has a synergistic effect when co-administered with anti-CTLA-4 antibodies, whereas the effect is less impressive when administered with anti-CD25 monoclonal antibodies. However, as with anti-CTLA-4 antibodies, autoimmune symptoms have been detected. At the time of writing human studies have yet to commence.

Imatinib (Glivec, Novartis UK, Frimley, Surrey, UK), a tyrosine kinase inhibitor, is the treatment of choice for advanced gastrointestinal stromal tumours. More recently in patients with chronic myeloid leukaemia treated with stem cell transplantation, imatinib was given to accentuate the graft-*vs*-leukaemia reaction. This resulted in a significant dose-dependent decrease in CD69 GITR, CTLA-4 and FOXP3 expression, and IL-10 and TGF-*β* secretion by Tregs (Chen *et al*, 2007). Though imatinib may not be the most suitable treatment for graft-*vs*-leukaemia reactions, it may be of benefit in Treg downregulation as an adjunct to cancer immunotherapy. Other tyrosine kinase inhibitors such a suntinib (effective in the treatment of RCC) also attenuate Treg numbers in mice (Hipp *et al*, 2007).

Bevicuzimab is an effective antibody at preventing tumour angiogenesis and works by preventing the activation of tyrosine kinase. In RCC patients treated with Bevacizumab a drop in Treg numbers was observed in clinical responders (Passalacqua *et al*, 2008). The actual mechanism that leads to Treg attenuation and depletion is yet to be determined.

A recent *in vitro* study showed the ability of lenalidomide (Revlimid, Celgene Corporation, Summit, NJ, USA; CC-5013) and CC-4047 to suppress Treg function and proliferation; effects that may be mediated by downregulation of FOXP3 (Galustian *et al*, 2008). Both drugs are active against multiple myeloma, and it is tempting to speculate that this is mediated by their anti-Treg properties, although co-stimulatory and anti-angiogenic activity have also been shown.

Fluorescence-activated cell sorting isolated human Tregs also seem to be sensitive to CD95-induced apoptosis, whereas isolated effector T cells seem to be resistant. This may be a method of ensuring that Tregs are eliminated in the acute phase of an immune response (Fritzsching *et al*, 2005).

Activation of toll-like receptor (TLR) pathways in dendritic cells confers resistance of naïve T cells to Treg-mediated suppression. TLR ligands also have a direct effect on Tregs, countering their suppressive properties without the need for dendritic cells. TLR 8 (present in high levels on Tregs) activation by its ligand RNA 40 (a specific oligoribonucleotide ) negated the suppressive function of Tregs in mice (Peng *et al*, 2005). Though TLR8 activation is mediated by the MyD88 intracellular pathway, its downstream effects, which ultimately lead to Treg suppression, require elucidation and potential effects on immunotherapy have yet to be determined.

Activation of OX40, a co-stimulatory molecule belonging to the TNF receptor superfamily, leads to expansion of CD4+ and CD8+ T cells resulting in tumour rejection in murine models (Weinberg *et al*, 2000). A recent murine study showed that OX40 activation not only reversed effector T-cell hyporesponsiveness, but also negated Treg function leading to tumour rejection (Piconese *et al*, 2008).

## Conclusion

The realisation that Tregs play a major role in the development of human cancer has led to the discovery that these cells are effective in shielding cancers from a potential immune response. Moreover, the dramatic effects of some conventional chemotherapeutic agents may not solely be because of a direct anti-cancer effect, but may also rely on an indirect and additive effect on the cellular immune response to cancer.

Here we focused on the potential clinical effects of immune modulation of Tregs in humans, as successful murine studies do not always translate to success in humans; possibly reflecting differences in Treg types and function between the two species. At present direct therapeutic effect on human Tregs are generally gauged by observing cell number and crude function, mainly in the peripheral blood. It has been argued that peripheral blood sampling does not reflect the most relevant compartment, and that monitoring the intratumoural environment through biopsy or fine-needle aspirates may provide more useful information about the effector functions of Tregs in cancer. Few studies delve into the tumour milieu to objectively assess effects on intratumoural Tregs. The study by Powell investigating the effect of LMB-2 on three patients with MM suggests that there is a concomitant depletion of Tregs peripherally and within the tumour. The numbers within this study are very small as serial tumour sampling from patients is not easy. Careful phase I and II studies will be required in future to ascertain any therapeutic or prognostic effect with more focus on real-time changes occurring within the tumour milieu.

With the availability of several new monoclonal antibodies and a reassessment of other treatments whose mechanisms are poorly understood, new protocols will be able to target Treg cells in order to make anti-cancer treatments more effective. At present, the best of method of Treg modulation is yet to be discerned and many questions are still unanswered as to which type of cells are most effective against tumours; however, the future of Treg modulation within the remit of cancer immunotherapy looks promising.

## Figures and Tables

**Table 1:** Phase I studies administering diftitox

**Study**	**Diftitox dosing strategy**	**Number of patients**	**CR**	**PR**	**SD**	**PD**	**Toxicity**
Earlier treated indolent non-Hodgkin's lymphoma (Kuzel *et al*, 2007)	Diftitox administered at 18 *μ*g kg^−1^ per day for 5 days every 21 days	29 (3 unevaluable)	0	3	14	9	Mainly grade I and II but also one fatal thrombolembolism
Combined with rituximab for relapsed/refractory B-cell non-Hodgkin's lymphoma (Dang *et al*, 2007)	Rituximab at a 375 mg m^–2^ on day 1 of each 3-week cycle i.v. diftitox 18 *μ*g kg^–1^ per day on days 2–6 of cycle	38	6	6	7	19	Mainly grade I and II toxicity but loss of visual acuity in two patients
Relapsed/refractory T-cell non-Hodgkin's lymphoma (Dang *et al*, 2007)	Diftitox 18 *μ*g kg^–1^ per day for 5 days every 3 weeks for up to eight cycles	27	6	7	7	7	Mainly grade I toxicity
Earlier treated chronic lymphocytic leukemia (Frankel *et al*)	Diftitox 5 days every 21 days at 18 *μ*g kg^–1^ per day for up to 8 cycles	22 tolerating >2 cycles	1	5	6	9	Moderate toxicities
Metastatic melanoma and renal-cell carcinoma (Attia *et al*)	Diftitox administered for 5 days every 21 days in seven patients at 9 *μ*g kg^–1^ per day, and in six patients at 18 μg kg^–1^ per day	12 metastatic melanoma, one metastatic renal-cell carcinoma	0	0	Not mentioned	Not mentioned	11 patients developed transient grade III/IV toxicity

**Table 2:** Phase I and II studies administering anti-CTLA-4 treatment

**Study**	**Disease**	**Number of patients**	**Dosing of anti-CTLA-4**	**Side effects**	**Objective response**
Phase I (Phan *et al*, 2008)	Metastatic melanoma	14	3 mg kg^–1^ (three times weekly)+gp100 melanoma-associated antigen	Six grade III/IV autoimmune toxicity	Three in total
Phase I study (Hodi *et al*, 2003)	Metastatic melanoma	7			Two tumour necrosis and two T-cell infiltration
	Ovarian	2	3 mg kg^–1^ (one dose)	Five grade I/II and I had IV grade autoimmune toxicity	One tumour necrosis and two reduction/stabilization of Ca-125
Phase I study (Sanderson *et al*, 2005)	Metastatic melanoma	19	0.3 mg kg^–1^+peptide immunizations (*n=*7)	0	Seven in total
			1.0 mg kg^–1^+peptide immunizations (*n=*7)	One grade III autoimmune toxicity	
			3.0 mg kg^–1^+peptide immunizations (*n=*5)	Three III autoimmune toxicity	
Phase I study (Attia *et al*, 2005b)	Metastatic melanoma	56	3 mg kg^–1^ ( three times weekly)+peptide vaccinations (*n=*29)	14 grade III/IV autoimmune toxicity	4
			3 mg kg^–1^ then 1 mg kg^–1^ (three times weekly)+ peptide vaccinations (*n=*27)		3
Phase I and II study (Maker *et al*, 2005a)	Metastatic melanoma	36	0.1 mg kg^–1^ + IL-2 (*n=*3)	Five grade III/IV autoimmune toxicity	0
			0.3 mg kg^–1^ + IL-2 (*n=*3)		
			1.0 mg kg^–1^ + IL-2 (*n=*3)		
			2.0 mg kg^–1^ + IL-2 (*n=*3)		3
			3.0 mg kg^–1^ + IL-2 (*n=*24)		5
